# Glycerol phenylbutyrate efficacy and safety from an open label study in pediatric patients under 2 months of age with urea cycle disorders

**DOI:** 10.1016/j.ymgme.2020.12.002

**Published:** 2020-12-23

**Authors:** Nicola Longo, George A. Diaz, Uta Lichter-Konecki, Andreas Schulze, Michal Inbar-Feigenberg, Robert L. Conway, Allison A. Bannick, Shawn E. McCandless, Roberto Zori, Bryan Hainline, Nicholas Ah Mew, Colleen Canavan, Thomas Vescio, Teresa Kok, Marty H. Porter, Susan A. Berry

**Affiliations:** a University of Utah, Salt Lake City, UT, USA; b Icahn School of Medicine at Mount Sinai, New York, NY, USA; c Children's Hospital of Pittsburgh, Pittsburgh, PA, USA; d Hospital for Sick Children, University of Toronto, Toronto, Ontario, Canada; e Wayne State University School of Medicine, Detroit, MI, USA; f University of Colorado Anschutz Medical Campus and Children's Hospital Colorado, Aurora, CO, USA; g University of Florida, Gainesville, FL, USA; h Indiana University School of Medicine, Indianapolis, IN, USA; i Children's National Medical Center, Washington, DC, USA; j Horizon Therapeutics plc, Deerfield, IL, USA; k University of Minnesota, Minneapolis, MN, USA

**Keywords:** Urea cycle disorder, Neonate, Glycerol phenylbutyrate, Pediatric, Hyperammonemia

## Abstract

**Background/Aims:**

Neonatal onset Urea cycle disorders (UCDs) can be life threatening with severe hyperammonemia and poor neurological outcomes. Glycerol phenylbutyrate (GPB) is safe and effective in reducing ammonia levels in patients with UCD above 2 months of age. This study assesses safety, ammonia control and pharmacokinetics (PK) of GPB in UCD patients below 2 months of age.

**Methods:**

This was an open-label study in UCD patients aged 0 – 2 months, consisting of an initiation/transition period (1 – 4 days) to GPB, followed by a safety extension period (6 months to 2 years). Patients presenting with a hyperammonemic crisis (HAC) did not initiate GPB until blood ammonia levels decreased to below 100 μmol/L while receiving sodium phenylacetate/sodium benzoate and/or hemodialysis. Ammonia levels, PK analytes and safety were evaluated during transition and monthly during the safety extension for 6 months and every 3 months thereafter.

**Results:**

All 16 patients with UCD (median age 0.48 months, range 0.1 to 2.0 months) successfully transitioned to GPB within 3 days. Average plasma ammonia level excluding HAC was 94.3 μmol/L at baseline and 50.4 μmol/L at the end of the transition period (p = 0.21). No patient had a HAC during the transition period. During the safety extension, the majority of patients had controlled ammonia levels, with mean plasma ammonia levels lower during GPB treatment than baseline. Mean glutamine levels remained within normal limits throughout the study. PK analyses indicate that UCD patients <2 months are able to hydrolyze GPB with subsequent absorption of phenylbutyric acid (PBA), metabolism to phenylacetic acid (PAA) and conjugation with glutamine. Plasma concentrations of PBA, PAA, and phenylacetylglutamine (PAGN) were stable during the safety extension phase and mean plasma phenylacetic acid: phenylacetylglutamine ratio remained below 2.5 suggesting no accumulation of GPB. All patients reported at least 1 treatment emergent adverse event with gastroesophageal reflux disease, vomiting, hyperammonemia, diaper dermatitis (37.5% each), diarrhea, upper respiratory tract infection and rash (31.3% each) being the most frequently reported.

**Conclusions:**

This study supports safety and efficacy of GPB in UCD patients aged 0 -2 months who cannot be managed by dietary protein restriction and/or amino acid supplementation alone. GPB undergoes intestinal hydrolysis with no accumulation in this population.

## 1. Introduction

Urea cycle disorders (UCDs) are rare inborn errors of metabolism caused by a defect in any of the enzymes or transporters required for nitrogen disposal, with an estimated incidence of at least 1:35,000 births [1]. They are characterized by the accumulation of toxic levels of ammonia and glutamine in the blood and brain, manifesting as encephalopathy and death if untreated [1]. Elevated ammonia concentrations can cause a broad spectrum of neurocognitive disturbances ranging from learning disabilities and problems with executive function to severe brain compromise [2]. Proximal defects of the urea cycle (ornithine transcarbamylase [OTC], carbamyl phosphate synthase [CPS], and *N*-acetyl-glutamate synthase [NAGS] deficiencies) tend to be more severe and present earlier when compared to distal UCDs (argininosuccinate synthase [ASS] and lyase [ASL] deficiencies) [1]. Complete enzyme deficiencies typically present with hyperammonemic crisis (HAC) shortly after birth, with high rates of mortality, cognitive impairment, and high risk of recurrence [3]. In contrast, partial deficiencies have onset at any age with more variable presentations, can cause neurocognitive deficits, and result in premature death [4].

Despite advances in technology and awareness about UCDs, newborn screening (NBS) cannot detect all UCD subtypes. The current recommended uniform screening panel (RUSP) in the U.S. includes ASS and ASL deficiencies, but not the proximal UCDs [5]. NBS allows early diagnosis and is beneficial to improve neurocognitive outcomes in patients with ASS and ASL deficiencies [6]. Patients with early onset UCDs may appear normal at birth, but become acutely ill, usually within a few days of life [1]. Nonspecific symptoms include refusal of food, vomiting, and lethargy that can worsen rapidly progressing to coma [7]. Because early symptoms in affected neonates are nonspecific, critically ill infants are frequently misdiagnosed, resulting in delayed treatment or death [3].

Therapeutic interventions include hemodialysis at time of acute presentation, in addition to provision of calories as carbohydrates and lipids and intravenous nitrogen scavenger. After stabilization, chronic therapy includes a low protein diet, arginine or citrulline supplements, and nitrogen scavengers, with severe cases benefitting from liver transplantation [1]. The goal of therapy is to improve survival, maintain metabolic control by removing excess nitrogen and, therefore, limit long-term sequelae such as cognitive impairment. Chronic nitrogen scavengers routinely utilized include sodium benzoate, sodium phenylbutyrate (NaPBA), and glycerol phenylbutyrate (GPB) or a combination. Benzoate conjugates with glycine to form hippuric acid and phenylbutyrate is converted to its active metabolite phenylacetic acid (PAA), which is conjugated with glutamine to form phenylacetylglutamine (PAGN) [8]. Both hippuric acid and PAGN are excreted in urine. In healthy volunteers, phenylacetate disposes of more nitrogen than benzoate [9], but comparative studies in patients with urea cycle disorders are limited [10,11].

Neurocognitive outcome depends on the type of urea cycle defect and on the height of the initial peak plasma ammonium concentration, which is inversely associated with neurocognitive outcomes in proximal UCDs [6]. Individuals with ASS and ASL deficiencies identified by NBS had better neurocognitive outcomes than those diagnosed after the manifestation of first symptoms. In OTC and ASS deficiencies, there is not much evidence that monoscavenger therapy with NaPBA is superior to sodium benzoate in providing cognitive protection [6]. Early liver transplantation appears to be beneficial for preserving neurocognitive outcome in UCD patients [6].

GPB was developed to address chronic compliance difficulties with NaPBA, such as salt content, taste, and dosing volume [12,13]. GPB is a liquid triglyceride formulation consisting of three molecules of phenylbutyrate (PBA) joined to a glycerol backbone (Supplementary Fig. 1) [14]. It is a nearly tasteless and odorless liquid, contains no sugar or sodium, and is administered in low volume doses [10,15]. GPB is currently approved (as Ravicti®) in the U.S. for the chronic management of adult and pediatric patients with UCDs that cannot be managed by dietary protein restriction and/or amino acid supplementation alone.

GPB has previously demonstrated safety and efficacy in numerous patient cohorts throughout its clinical trial program [16-23]. It was first approved for patients 2 years of age and older in 2013, 2 months of age and older in 2017, and finally for newborn patients and older in 2018. Prior to this study, while in vitro data suggested that pancreatic enzymes capable of hydrolyzing GPB were present in newborns, it was not known if these enzymes were sufficiently mature to digest GPB [24]. This study was conducted to fulfill the Food and Drug Administration (FDA) post-marketing requirement and a Pediatric Investigation Plan requirement by the European Medicines Agency's (EMA) Pediatric Committee to assess GPB treatment in UCD patients <2 months of age. This study also served to confirm the safety of GPB in this patient population with regards to accumulation and excretion of GPB analytes. This report provides short- and long-term evidence of efficacy, safety, and pharmacokinetics (PK) in pediatric UCD patients <2 months of age.

## 2. Materials and methods

### 2.1. Study design

This was an open-label study consisting of a transition period to GPB, followed by a safety extension period where patients received GPB for at least 6 months and up to 2 years of GPB treatment (NCT 02246218). Study patients included newborns to infants <2 months of age with either a diagnosed or clinically suspected UCD (all types, except NAGS deficiency). A UCD diagnosis was suspected when a patient experienced a hyperammonemic event with ammonia level >100 μmol/L accompanied by signs and symptoms compatible with hyperammonemia in the absence of other obvious causes. The UCD diagnosis was confirmed with genetic testing within 60 days of study entry. If genetic testing was inconsistent with or excluded a UCD diagnosis, the patient was withdrawn from the study. After enrollment, initiation or transition to GPB occurred from Day 1 to Day 4, followed by a monitoring period of approximately 24 h. Patients presenting with a HAC did not initiate GPB until blood ammonia levels decreased to below 100 μmol/L while receiving sodium phenylacetate/sodium benzoate injection and/or hemodialysis.

Patients' ammonia levels, PK analytes, and safety were evaluated during transition (up to 7 days of GPB treatment), monthly during the safety extension for 6 months, and then every 3 months thereafter (Schedule of assessments, Supplementary Table 1). Baseline ammonia was defined as the mean of ammonia values within 7 days prior to Day 1 of GPB dosing. Appropriate limitations for blood sampling in infants and neonates were followed and in cases where the blood limits were met, blood samples were collected in the following order of priority: (1) ammonia samples; (2) PK samples; (3) safety samples (chemistry and hematology); and (4) amino acid samples.

### 2.2. Study drug

Ravicti® contains 1.1 g/mL of GPB which delivers 1.02 g/mL of phenylbutyrate [25]. GPB was administered orally via syringe, just prior to breastfeeding or intake of formula or food. If necessary, GPB was added to a small amount of formula in a small syringe, nipple, or other dosing device and administered orally immediately prior to feeding. A gastric or nasogastric tube was used for patients unable to tolerate oral dosing. The recommended dosing regimen was 3–6 times per day depending on the feeding schedule and at the discretion of the investigator. The starting dose of GPB was based on UCD status (newly diagnosed or already stable on NaPBA and/or sodium benzoate) and whether a HAC was present (Table 1). As part of the protocol, GPB treatment was stopped if patients underwent liver transplantation.

### 2.3. Endpoints

The primary efficacy endpoint was successful transition to GPB with controlled ammonia (defined as no clinical symptoms of hyperammonemia and ammonia <100 μmol/L). The investigator made an assessment of clinical status at the end of the transition period and determined if successful transition to GPB occurred during the period.

Safety endpoints were HACs during the first 6 months on GPB, treatment emergent adverse event (TEAE) rate, amino acid panel, development and growth assessed as *Z*-scores for height, head circumference, weight, body mass index (BMI) and body surface area (BSA), standard clinical laboratory tests and amino acid assessments, concomitant medications, vital signs, and physical examinations. Height (length), weight, and head circumference were measured twice at each scheduled assessment, and an average was calculated.

The PK evaluation of GPB (including plasma PBA, PAA, and PAGN) was performed to establish the effectiveness of intestinal hydrolysis of GPB and the rate at which PBA was converted to urinary PAGN and to establish the efficiency of conjugation of PAA and glutamine.

### 2.4. Statistical analysis

All analyses and tabulations were performed using SAS® version 9.2 or above. Data were summarized using descriptive statistics (number of subjects [n], mean, standard deviation [SD], median, minimum, and maximum) for continuous variables and frequency and percentages for categorical variables. A Wilcoxon signed-rank test was performed for the change from baseline to end of transition for ammonia levels. Summaries were presented by age (<1 month, 1 month to <2 months, and birth to <2 months). Note that the age (days) is derived as Date of Screening Visit – Date of Birth with 30 days being used as 1 month when defining age categories.

Two analysis populations were defined for this study:

- Safety population (for all efficacy and safety analyses): All patients who received any amount of study drug.
- PK evaluable population: All patients from the Safety population with individual concentration-time profiles (i.e., measurable concentrations for PBA, PAA, or PAGN) that allowed computation of meaningful PK parameter values.

To conduct a population PK analysis of GPB in this age group, empirical Bayes estimates (using software package NONMEM™, version 7.2) of the individual PK parameters of oral clearance (CL/F) and volume of distribution (V/F) were generated based on the individual plasma concentration measurements using the PK model previously described [19]. These individual PK parameters were then allometrically scaled to address effects of body size. The plasma concentrations that were below the lower limit of quantitation (LLOQ) were set to one-half of the LLOQ for each analyte.

## 3. Results

### 3.1. Patient disposition and demographics

**Fig. 1.**
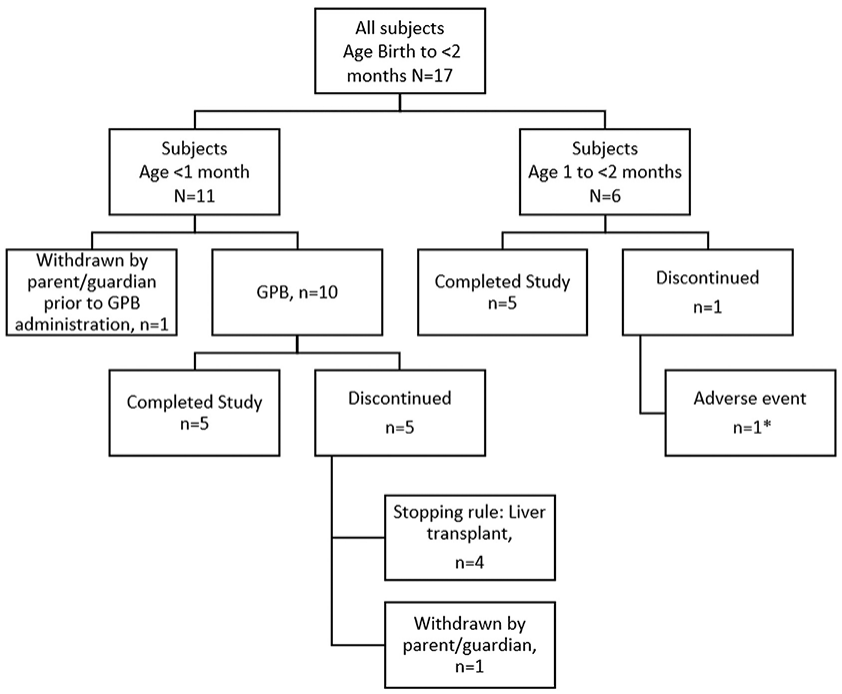
Patient disposition. No patients discontinued during the transition to GPB. *patient had non-serious adverse event (elevated liver enzyme) leading to discontinuation.

Seventeen patients originally enrolled in the study, 16 of whom received at least one dose of study drug and were included in the Safety population (Fig. 1). With the exception of one patient who withdrew prior to dosing, all patients (16 [94.1%]) completed through at least Month 1, 15 (88.2%) completed through at least Month 3, 10 (58.8%) completed through at least Month 6, and 3 (17.6%) completed between 18 and 24 months (Supplementary Fig. 2). The longest duration on study for a patient was 622 days.

Patient demographics are presented in Supplementary Table 2. Median age was 0.48 months (mean [±SD] 0.83 ± 0.697 months; range 0.1 to 2.0 months). The exposure to GPB during transition is summarized in Supplementary Table 3.

Patient characteristics are presented in Table 2. Just over one-half of the patients were male (9 [56.3%]) and a majority were diagnosed with OTC or ASS deficiency. Onset of UCD for most patients was neonatal (93.8%). Seven patients (43.8%), 5 of whom were 1 to <2 months old, were diagnosed by newborn screening. The most frequent UCD treatments prior to the study were the following (note that patients could have received more than 1 type of treatment): low protein diet (87.5%), dietary supplements (81.3%), and/or sodium phenylbutyrate powder (62.5%). One-half of patients had a history of gastric/nasogastric/nasojejunal tube use. Prior to the start of the study, 10 patients were stable on NaPBA, 3 patients were newly diagnosed and not in HAC, and 3 patients were in HAC and receiving intravenous sodium phenylacetate/sodium benzoate.

### 3.2. Ammonia and glutamine

**Fig. 2.**
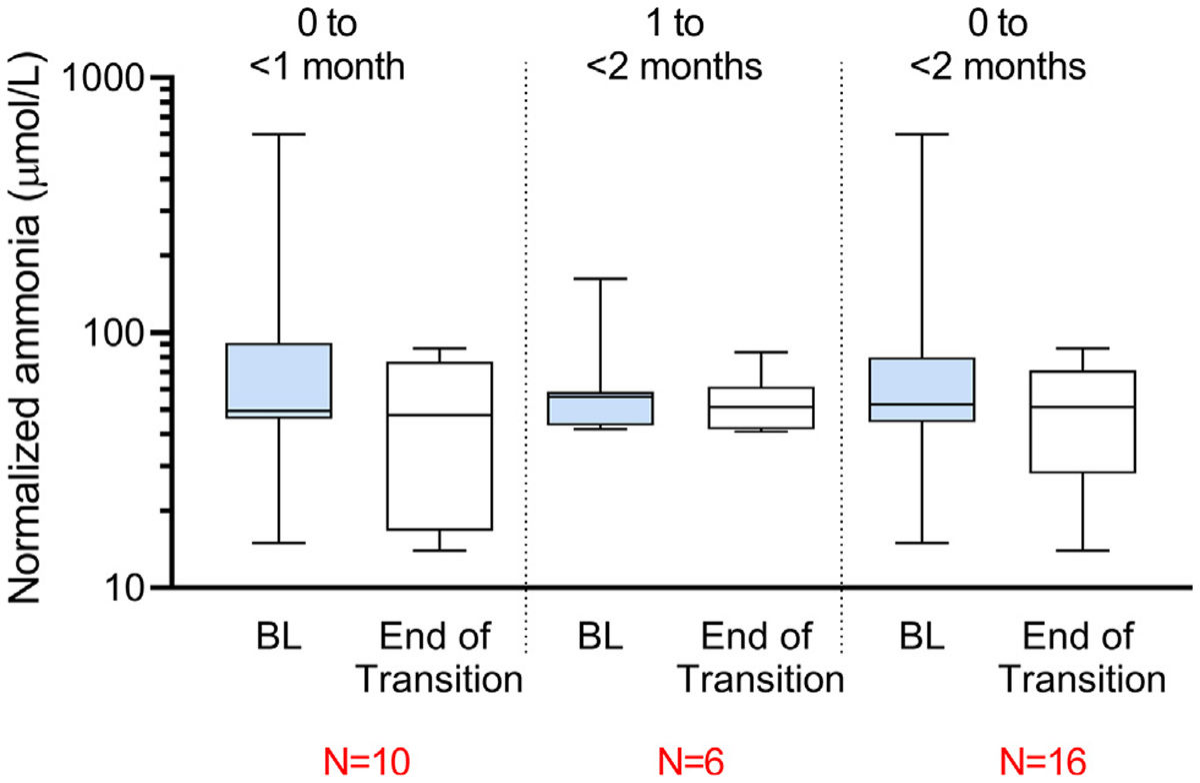
Box and whisker plots of normalized ammonia levels (log-scale) at baseline and end of the transition in patients with urea cycle disorders. Box represents inter-quartile range and the horizontal line within the box represents the median ammonia level. The whiskers ends represent the range and are grouped for age at enrollment (indicated in the figure). None of the comparisons between baseline and end of transition were statistically significant. Ammonia levels were normalized to a common upper limit of normal (ULN) for the analyses. All data were converted to SI units before normalization. The conversion formula was μg/dL × 0.5872 = μmol/L. The standard normal reference range for this study was 28–57 μmol/L Normalization was done with the formula s = x*(Us/Ux) where s was the normalized laboratory value, x was the original laboratory value, Ux was the upper limit of normal from the original laboratory and Us was the upper limit of normal for the standard laboratory.

Ammonia levels at baseline and at the end of the transition period for 0 to <1 month, 1 to <2 months, and the overall population (0 to <2 months) are depicted in Fig. 2. Transition occurred when the following conditions were met: no signs and symptoms of hyperammonemia, ammonia level less than 100 μmol/L, and eligibility for discharge per Investigator judgment. All patients achieved successful transition by the end of 3 days of receiving GPB alone. Average plasma ammonia level among all GPB patients was 94.3 μmol/L at baseline and 50.4 μmol/L at the end of the transition period (*p* = 0.21).

**Fig. 3.**
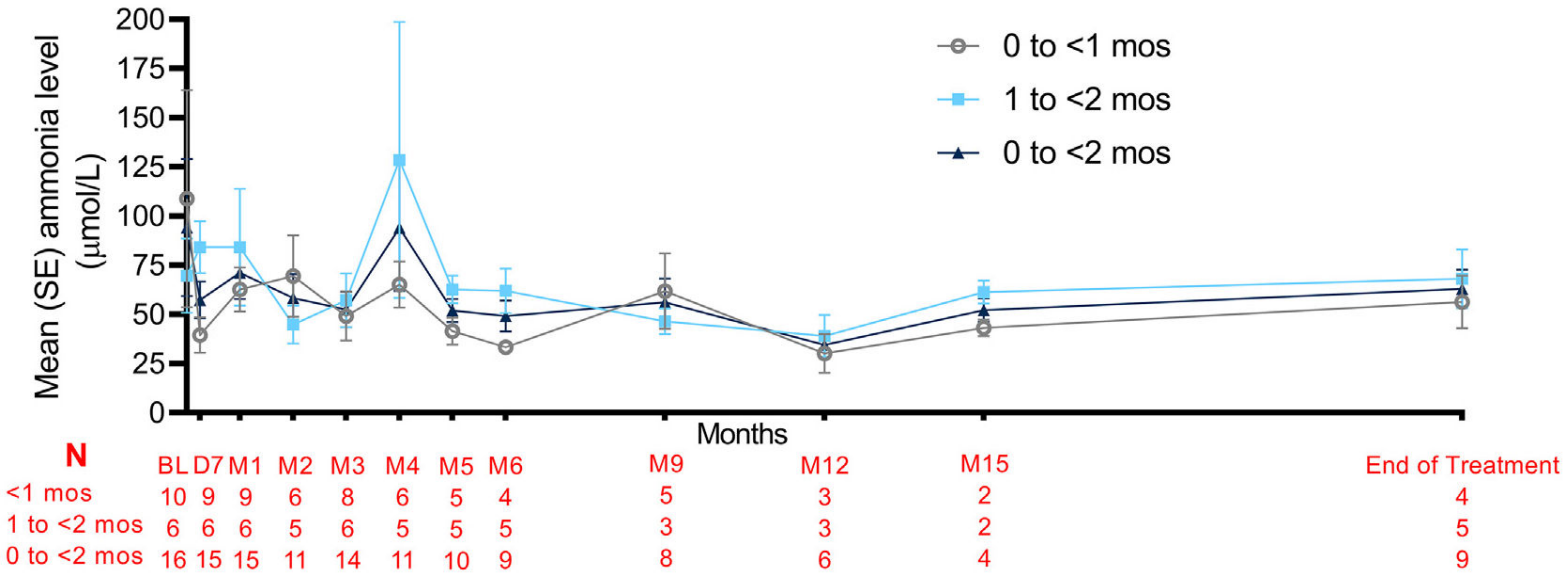
Mean normalized ammonia levels in patients with UCD during the safety extension period. Patients continued to receive GPB after the transition period with the ammonia levels indicated. The number of patients at each time point is indicated below the figure. Data are shown as averages ± SE. Month 4 levels in the 1 to <2 month group was skewed by 1 patient who was not administered GPB for 36 days prior to the drawing of this ammonia level and who ultimately discontinued GPB due to a TEAE of elevated liver enzymes (Grade 1). Ammonia levels were normalized as described in the legend to Fig. 2. Month 18 levels were only available for 3 patients and therefore excluded.

The transition period was followed by a safety extension period (Fig. 3) where patients received GPB until they reached 2 years or until they had received GPB in the study for at least 6 months and were eligible for commercially available drug. The majority of patients had controlled ammonia levels during the first 12 months of the safety extension period. There were fewer data points after 12 months due to patient discontinuations and missing data.

**Fig. 4.**
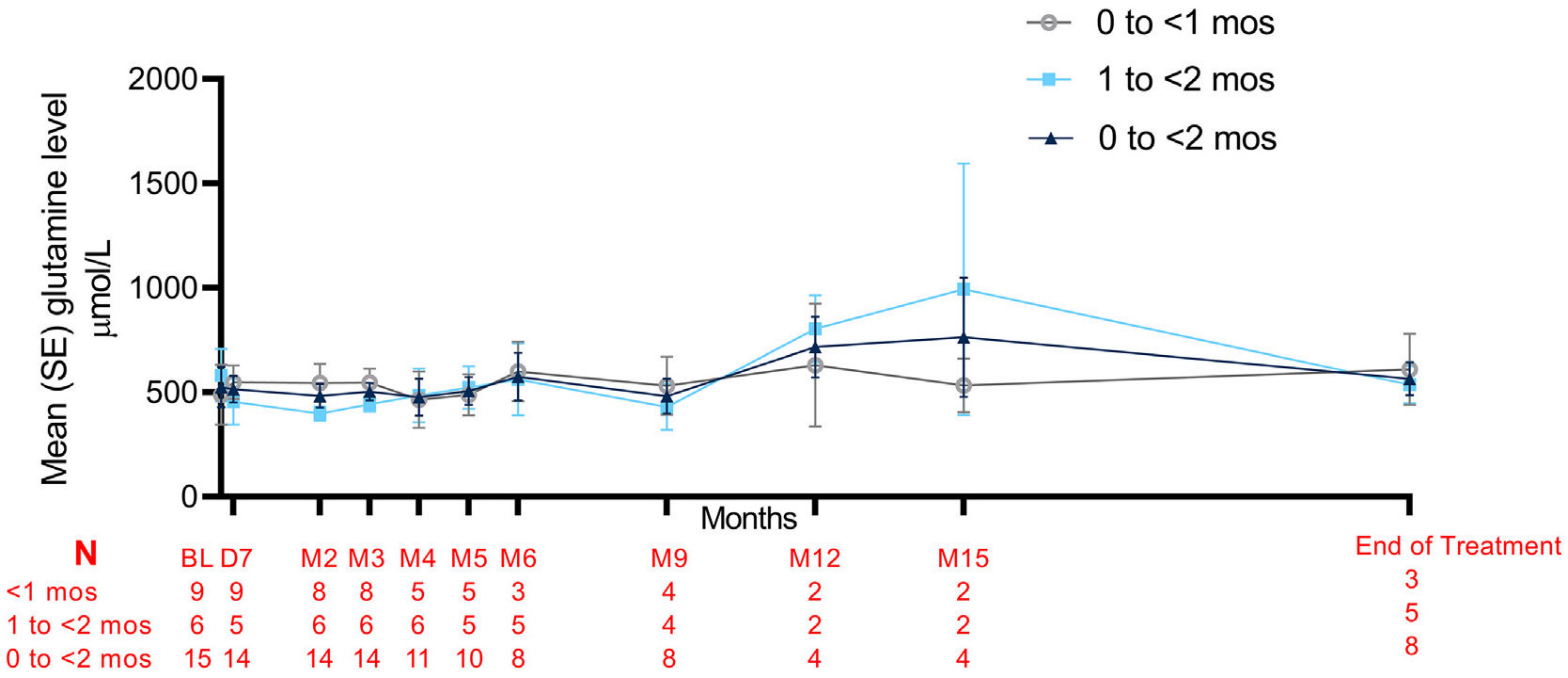
Mean (SE) glutamine levels in 0 to <2 month-old patients with urea cycle disorders. Glutamine levels were normalized to upper limit of normal of 950 μmol/L in the same way as ammonia levels. Month 18 levels were only available for 2 patients and therefore excluded.

UCD guidelines suggest that glutamine levels not exceeding 1000 μmol/L are considered tolerable in patients with urea cycle disorders [3]. Mean glutamine levels remained <1000 μmol/L on average throughout the study (Fig. 4), although the small number of patients at each time point from Month 6 on precludes any meaningful analyses.

### 3.3. Phenylbutyrate metabolites

A total of 140 plasma PBA, PAA, and PAGN concentrations from 16 patients (10 patients in 0 to <1 month cohort and 6 patients in the 1 to <2 months cohort) were included in the population PK analysis.

**Fig. 5.**
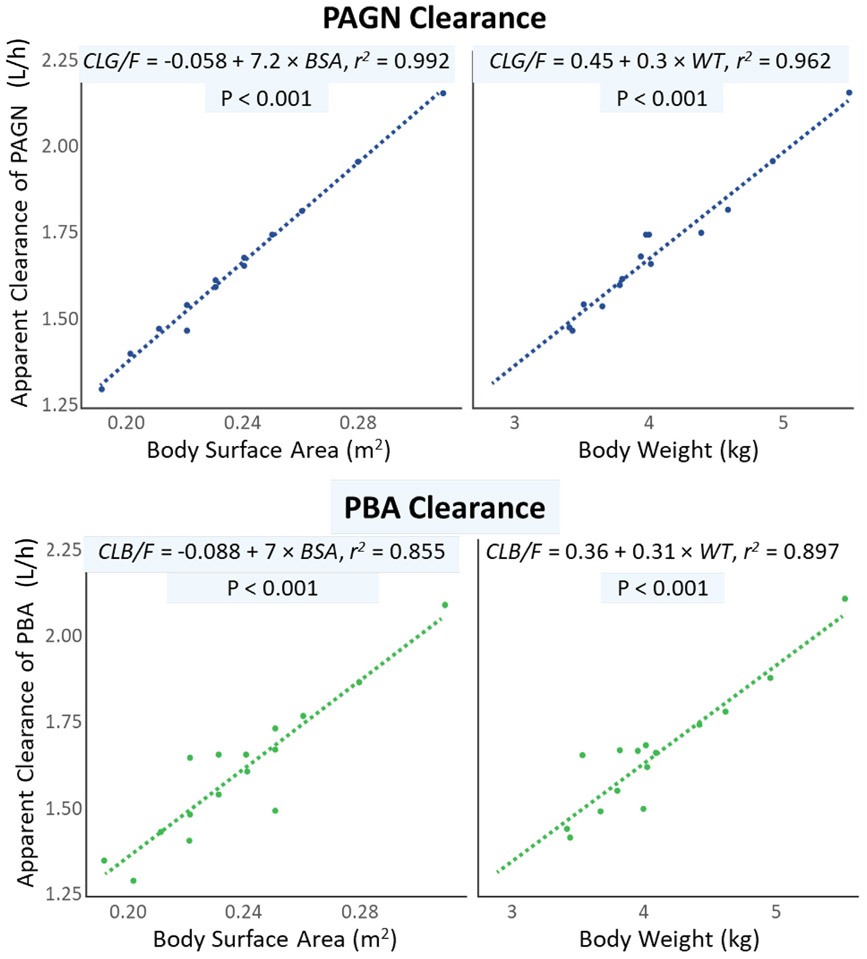
Correlation between apparent clearance (geometric mean estimate) of (PBA and PAGN with body surface area (left) and weight (right) in 0 to <2 month-old patients with urea cycle disorders. BSA and body weight were significant predictors of PBA and PAGN clearance in patients 0 to <2 months of age. Two extremely high PAGN levels were beyond 5 times the SD, were considered artifacts and not included in the PK analysis. Abbreviations: PBA, phenylbutyrate; PAGN, phenylacetylglutamine; BSA, body surface area.

Clearance and volume of distribution of GPB-associated analytes increased with age and body size (Fig. 5). Comparable correlations of PBA and PAGN clearance with weight and BSA support the use of both BSA or weight-based dosing in patients 0 to <2 months of age with UCDs.

Substantial inter-individual variability in analyte concentrations was observed in the 0 to <2 month-old population. All three analytes (PBA, PAA, and PAGN) were consistently measurable with most below limit of quantification (BQL) at the pre-dose measurement, indicating that there was intestinal hydrolysis of GPB with subsequent absorption of PBA, metabolism to PAA, and conjugation with glutamine (Supplementary Table 4). Plasma concentrations of PBA, PAA, and PAGN were stable during the safety extension phase (Supplementary Table 5). PAGN concentrations in the urine ranged from 166 to 14,500 μg/mL on the first full day of GPB treatment. There was a general increase in PAGN excretion in urine over time. The presence of expected metabolites demonstrates intestinal hydrolysis of GPB and subsequent absorption of PBA with metabolism to PAA and conjugation with glutamine. Fig. 1 presents the mean PAA:PAGN ratio by clinic visit for all patients under the age of 2 months enrolled in the trial. The PAA:PAGN ratio was proposed to be a useful biomarker in a random blood draw to identify patients at risk of high PAA levels [26]. Reversible neurological adverse events were previously reported in cancer patients receiving intravenous PAA; these patients had PAA plasma levels >500 μg/dL [27]. Mokhtarani et al. investigated the relationship between PAA levels and neurological adverse events in patients (with UCD and hepatic encephalopathy) treated with GPB and did not find a relationship, although transient adverse events like headache and nausea were observed in healthy adults that correlated with PAA levels [26].

### 3.4. Hyperammonemic crises and adverse events

Prior to enrollment, 6 of 16 patients (0 to 2 month group) had 1 HAC, and 1 patient had 2 crises. During the transition period, no patients had a HAC. During the safety extension, 3 patients had 1 HAC and 2 patients had 2 crises each, all of which occurred in patients <1 month of age. There were 5 additional patients who had at least one ammonia level that exceeded 100 μmol/L, that were not associated with a HAC (there were no symptoms).

Most frequently reported TEAEs were gastroesophageal reflux disease, vomiting, hyperammonemia, diaper dermatitis (37.5% each), diarrhea, upper respiratory tract infection, and rash (31.3% each)(Table 3). Of note, of the 6 patients who experienced a TEAE of hyperammonemia, one was on concomitant sodium phenylbutyrate and another was on concomitant sodium benzoate. All patients reported at least 1 TEAE. One patient had a TEAE of leukocytosis and 1 patient had a TEAE of lymphocytosis. Ten patients (62.5%) reported at least 1 TEAE that was considered by the Investigator to be related to GPB. Eleven patients had at least 1 serious adverse event, none of which were considered related to the study drug by the investigator. There were no fatal TEAEs. One patient withdrew due to a serious adverse event (elevated liver enzymes).

### 3.5. Growth and development

Actual height, weight, BMI, BSA, and head circumference for each patient increased over the study period. In order to determine how these baseline parameters compare to a standard reference population, Z scores were calculated. For all 16 patients aged 0 to 2 months, there was a mean decrease in *Z*-scores for weight, height, and head circumference at most safety extension visits and a mean increase in Z-scores for BSA and BMI (Supplementary Table 6). There was no apparent trend in these measurements.

## 4. Discussion

Children with early onset UCD require strict ammonia control and dietary protein restriction [28]. NaPBA is used as a nitrogen scavenger for newborn patients with UCDs although patients have historically reported difficulties taking it due to tolerance issues with vomiting and reflux [12,13,29]. Sodium benzoate is also utilized, although data is scarce and based on anecdotal evidence. To our knowledge, this is the first clinical study to investigate the use of a chronic nitrogen scavenger for UCD patients in the first two months of life and provides empirical evidence for UCD management.

Prior clinical studies in UCD patients treated with either GPB or NaPBA have demonstrated that the rate of conversion of PAA to PAGN varies directly with body size, such that it occurs more slowly in young children [14]. In this study, PK analyses found stable plasma concentrations of PBA, PAA, and PAGN during the safety extension phase. There was a general increase in PAGN excretion in urine over time, and no accumulation of PAA over the course of the study, demonstrating intestinal hydrolysis of GPB and subsequent metabolism to PAA and conjugation with glutamine in this cohort. The ratio of PAA to PAGN may help to optimize dose adjustment decisions. Unlike plasma PAA, the PAA-to-PAGN ratio is relatively constant over 24 h and is not affected by timing of the blood draw [26].

Infants were safely switched from NaPBA to GPB while maintaining control of ammonia and glutamine. While a TEAE of hyperammonemia was reported in 37.5% of patients, none of these events were fatal or deemed related to therapy. Maintaining ammonia control and preventing HACs in this fragile patient population improves survival, growth, development, and neurological outcomes [3]. The likely reason for better ammonia control was the improved tolerability of GPB as compared to existing therapies. All 16 (100%) patients had at least 1 TEAE, the majority being mild or moderate in severity. The TEAEs reported here are all events reported by the Investigators and are not necessarily considered GPB-related adverse drug reactions. Overall, the safety profile in this study is consistent with previous findings from GPB's clinical trial program, demonstrating the safety of GPB for management of UCD in various patient cohorts [16,30].

In this study, patients in the 0 to 1 month group included a higher percentage of patients with OTC deficiency who were more likely diagnosed symptomatically based on a higher baseline ammonia level; whereas the 1 to 2 month group included more ASS and ASL deficiencies, mostly identified by NBS with lower baseline ammonia levels. The degree of improvement in ammonia levels at end of transition (compared to baseline) was clearly more robust in the 0 to 1 month group, implying that there was potentially a distinct difference in the degree of metabolic liability between these 2 groups. This is not surprising, as patients with distal UCDs, especially those inclusive of the NBS population, tend to have milder disease courses less prone to hyperammonemia than the average proximal UCD patient. While some patients with “milder” disease may not need nitrogen scavenging therapy, studies have suggested that nitrogen scavenging therapy may confer some long-term neurocognitive benefits [31]. However, this was not evaluated in this study and represents a limitation to the data provided.

Significant improvements in the medical management of UCD patients have occurred over the past few decades with the optimization of low protein diets, nitrogen scavengers (sodium benzoate, NaPBA, and GPB), amino acid supplements, and hemodialysis. However, neonatal and early onset UCD patients are still at significant risk of death and morbidity. There are numerous novel therapeutic approaches currently being investigated, [32] including adeno-associated virus gene therapy, ornithine transcarbamylase deficiency mRNA therapy, enzyme replacement, and microbiome-based therapies that may come to fruition in the years ahead. In addition, liver transplant remains the therapy of choice in patients with severe UCDs [33]. GPB presents another option in infants to reduce the number of hyperammonemic events and improve clinical outcomes in children who are candidates for transplant in addition to providing a lifelong, acceptable therapy in children with milder UCDs.

## 5. Conclusions

This is the first study on safety and efficacy of nitrogen scavenger treatment in neonates. Our study demonstrates that GPB undergoes intestinal hydrolysis with no accumulation of PAA in UCD patients below 2 months of age. Patients aged 0 to 2 months are able to transition/initiate GPB and maintain controlled ammonia in the long term. The findings from this study support the use of GPB in UCD patients aged 0 to 2 months who cannot be managed by dietary protein restriction and/or amino acid supplementation alone.

## Supplementary Material

Suppl Materials

## Figures and Tables

**Fig.6.**
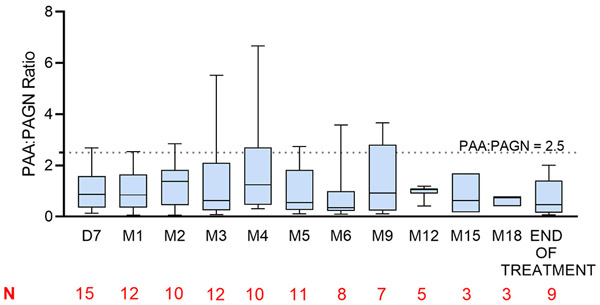
Phenylacetic acid: phenylacetylglutamine ratio in 0 to <2 month-old patients with urea cycle disorders. Ratios are reported for the safety extension trial. Data are presented as box and whiskers plots. Box represents inter-quartile range and the horizontal line within the box represents the median phenylacetic acid: phenylacetylglutamine ratio. The whiskers ends represent the range. A PAA:PAGN ratio of 2.5 (as indicated by the horizontal dotted line) is thought to identify patients at risk for PAA level > 500 μg/dL. Abbreviations: PAA, phenyl acetic acid; PAGN, phenylacetylglutamine

**Table 1:** Initial GPB doses used (divided in 3–6 doses per day, depending on feeding schedule and at the discretion of the physician).*

**Newly diagnosed patients presenting in hyperammonemic crisis (transitioning from intravenous sodium phenylacetate/sodium benzoate)** ^a^
Starting dose: 11.2 mL/m^2^/day
**Newly diagnosed patients not presenting in hyperammonemic crisis** ^a^
Starting dose: 8.5 mL/m^2^/day
**Patients on sodium phenylbutyrate** ^b^
An equivalent dose of GPB was calculated from the dose of sodium phenylbutyrate:
• Total daily dose GPB (mL) = grams of sodium phenylbutyrate powder × 0.81
• Total daily dose GPB (mL) = grams of sodium phenylbutyrate tablet × 0.86
**Patients on a stable dose of sodium benzoate** ^c^
• Total daily dose of GPB (mL) = grams of sodium benzoate × 0.5

* Doses were adjusted to achieve an ammonia level less than the upper limit of normal. Abbreviations: GPB, glycerol phenylbutyrate.

a Selected dose within manufacturer's dose range.

b Conversion to deliver the same amount of phenylbutyric acid.

c Conversion based on the fact that 2 mol of nitrogen are excreted in the urine with each mole of PBA administered as Ravicti® and 1 mol of nitrogen is excreted with each mole of hippuric acid derived from sodium benzoate

**Table 2:** Characteristics of 0 to <2 month-old patients with urea cycle disorders in this study.

	Age < 1 month (n = 10)	Age 1 month to <2 months (n = 6)	Total, Age 0 to< 2 months (*N* = 16)
Gender			
Male	7 (70%)	2 (33.3%)	9 (56.3%)
Female	3 (30%)	4 (66.7%)	7 (43.7%)
UCD diagnosis^a^			
OTCD	7 (70%)	1 (16.7%)	8 (50%)
ASSD	2 (20%)	5 (83.3%)	7 (43.8%)
ASLD	1 (10%)	0	1 (6.3%)
Onset			
Neonatal (<30 days of age)	10 (100%)	5 (83.3%)	15 (93.8%)
Infantile (>30 days to ≤2 years)	0	1 (16.7%)	1 (6.3%)
UCD treatment^b^			
Low protein diet^c^	8 (80%)	6 (100%)	14 (87.5%)
Dietary supplements	7 (70%)	6 (100%)	13 (81.3%)
Arginine	6 (60%)	5 (83.3%)	11 (68.8%)
Citrulline	6 (60%)	1 (16.7%)	7 (43.8%)
Nitrogen scavenging therapy for chronic management of UCD	5 (50%)	5 (50%)	10 (62.5%)
Sodium phenylbutyrate	5 (50%)	5 (83.3%)	10 (62.5%)
Sodium benzoate	0 (0%)	0 (0%)	0 (0%)
Therapy for acute	6 (60%)	2 (33.3%)	8 (50.0%)
hyperammonemia			
Sodium phenylacetate and sodium benzoate injection (Ammonul®)	5 (50%)	2 (33.3%)	7 (43.8%)
Other^d^	1 (10%)	0	1 (6.3%)
G/NG/NJ tube use^e^	5 (50%)	3 (50%)	8 (50%)

Abbreviations: ASLD, argininosuccinate lyase deficiency; ASSD, argininosuccinate synthase deficiency; G/NG/NJ, gastric/nasogastric/nasojejunal; OTCD, ornithine transcarbamylase deficiency; UCD, urea cycle disorder.

a There were no patients with carbamyl phosphate synthase deficiency, arginase deficiency, hyperornithinemia-hyperammonemia-homocitrullinuria syndrome, or citrin deficiency.

b Percentages may add up to more than 100% because more than 1 option could apply to the subject.

c Based on protein and calorie intake determined by the Investigator.

d Arginine hydrochloride.

e Not all subjects with a G/NG/NJ tube had GPB administered through the tube.

**Table 3:** TEAEs reported in ≥10% of 0 to <2 month-old patients with urea cycle disorders receiving glycerol phenylbutyrate.*

Most commonly reported TEAEs, *N* = 16	n (%)
Gastroesophageal reflux disease	6 (37.5)
Vomiting	6 (37.5)
Hyperammonemia	6 (37.5)
Dermatitis diaper	6 (37.5)
Diarrhea	5 (31.3)
Rash	5 (31.3)
Upper respiratory tract infection	5 (31.3)
Cough	4 (25.0)
Nasopharyngitis	4 (25.0)
Anemia	3 (18.8)
Dehydration	3 (18.8)
Flatulence	3 (18.8)
Teething	3 (18.8)
Ear infection	3 (18.8)
Urinary tract infection	3 (18.8)
Constipation	2 (12.5)
Pyrexia	2 (12.5)
Lethargy	2 (12.5)
Plagiocephaly	2 (12.5)
Increased hepatic enzymes	2 (12.5)
Oral candidiasis	2 (12.5)
Respiratory syncytial virus infection	2 (12.5)
Metabolic acidosis	2 (12.5)
Nasal congestion	2 (12.5)
Oropharyngeal pain	2 (12.5)
Thrombocytosis	2 (12.5)
Thrombocytopenia	2 (12.5)
Neutropenia	2 (12.5)

* Investigator-reported.

## References

[1] Ah Mew N, Simpson KL, Gropman AL, Urea cycle disorders overview. 2003 4 29 [Updated 2017 Jun 22]. In: Adam MP, Ardinger HH, Pagon RA, et al., editors. GeneReviews® [Internet]. Seattle (WA): University of Washington, Seattle; 1993–2020. Available from: https://www.ncbi.nlm.nih.gov/books/NBK1217/20301396

[2] Braissant O, McLin VA, Cudalbu C, Ammonia toxicity to the brain, J. Inherit. Metab. Dis 36 (4) (2013) 595–612. 23109059 10.1007/s10545-012-9546-2

[3] Häberle J, Burlina A, Chakrapani A, , Suggested guidelines for the diagnosis and management of urea cycle disorders: first revision, J. Inherit. Metab. Dis 42 (6) (2019) 1192–1230. 30982989 10.1002/jimd.12100

[4] Batshaw ML, Msall M, Beaudet AL, Trojak J, Risk of serious illness in heterozygotes for ornithine transcarbamylase deficiency, J. Pediatr 108 (2) (1986) 236–241. 3944708 10.1016/s0022-3476(86)80989-1

[5] Merritt JL 2nd, Brody LL, Pino G, Rinaldo P, Newborn screening for proximal urea cycle disorders: current evidence supporting recommendations for newborn screening, Mol. Genet. Metab 124 (2) (2018) 109–113. 29703588 10.1016/j.ymgme.2018.04.006

[6] Posset R, Gropman AL, Nagamani SCS, , Impact of diagnosis and therapy on cognitive function in urea cycle disorders, Ann. Neurol 86 (1) (2019) 116–128. 31018246 10.1002/ana.25492 PMC6692656

[7] Cohn RM, Roth KS, Hyperammonemia, bane of the brain, Clin. Pediatr. (Phila) 43 (8) (2004) 683–689. 15494874 10.1177/000992280404300801

[8] De Las Heras J, Aldamiz-Echevarria L, Martinez-Chantar ML, Delgado TC, An update on the use of benzoate, phenylacetate and phenylbutyrate ammonia scavengers for interrogating and modifying liver nitrogen metabolism and its implications in urea cycle disorders and liver disease, Expert Opin. Drug Metab. Toxicol 13 (4) (2017) 439–448. 27860485 10.1080/17425255.2017.1262843 PMC5568887

[9] Nagamani SCS, Agarwal U, Tam A, , A randomized trial to study the comparative efficacy of phenylbutyrate and benzoate on nitrogen excretion and ureagenesis in healthy volunteers, Genet. Med 20 (7) (2018) 708–716. 29693650 10.1038/gim.2017.167 PMC5924481

[10] Enns GM, Nitrogen sparing therapy revisited 2009, Mol. Genet. Metab 100 (2010) S65–S71. 20202877 10.1016/j.ymgme.2010.02.007

[11] Maestri NE, Brusilow SW, Clissold DB, Bassett SS, Long-term treatment of girls with ornithine transcarbamylase deficiency, N. Engl. J. Med 335 (12) (1996) 855–859. 8778603 10.1056/NEJM199609193351204

[12] Le Mons C, Lee B, Salehizadeh B, , Drugs for rare diseases: ravicti for urea cycle disorders as a case study, Wharton Healthcare Quart. 2 (3) (2013) 15–22.

[13] Mistry PK, Rare disease clinical research network’s urea cycle consortium delivers a successful clinical trial to improve alternate pathway therapy, Hepatology (Baltimore, Md) 57 (6) (2013) 2100–2102. 10.1002/hep.26106 23080135

[14] Monteleone JP, Mokhtarani M, Diaz GA, , Population pharmacokinetic modeling and dosing simulations of nitrogen-scavenging compounds: disposition of glycerol phenylbutyrate and sodium phenylbutyrate in adult and pediatric patients with urea cycle disorders, J. Clin. Pharmacol 53 (7) (2013) 699–710. 23775211 10.1002/jcph.92 PMC3923458

[15] Cederbaum S, Lemons C, Batshaw ML, Alternative pathway or diversion therapy for urea cycle disorders now and in the future, Mol. Genet. Metab 100 (3) (2010) 219–220. 20462778 10.1016/j.ymgme.2010.04.008

[16] Berry SA, Longo N, Diaz GA, , Safety and efficacy of glycerol phenylbutyrate for management of urea cycle disorders in patients aged 2 months to 2 years, Mol. Genet. Metab 122 (3) (2017) 46–53. 28916119 10.1016/j.ymgme.2017.09.002

[17] Diaz GA, Krivitzky LS, Mokhtarani M, , Ammonia control and neurocognitive outcome among urea cycle disorder patients treated with glycerol phenylbutyrate, Hepatology 57 (6) (2013) 2171–2179. 22961727 10.1002/hep.26058 PMC3557606

[18] Lee B, Rhead W, Diaz GA, , Phase 2 comparison of a novel ammonia scavenging agent with sodium phenylbutyrate in patients with urea cycle disorders: safety, pharmacokinetics and ammonia control, Mol. Genet. Metab 100 (3) (2010) 221–228. 20382058 10.1016/j.ymgme.2010.03.014 PMC2905228

[19] Lichter-Konecki U, Diaz GA, Merritt JL 2nd, , Ammonia control in children with urea cycle disorders (UCDs); phase 2 comparison of sodium phenylbutyrate and glycerol phenylbutyrate, Mol. Genet. Metab 103 (4) (2011) 323–329. 21612962 10.1016/j.ymgme.2011.04.013 PMC4880058

[20] Smith W, Diaz GA, Lichter-Konecki U, , Ammonia control in children ages 2 months through 5 years with urea cycle disorders: comparison of sodium phenylbutyrate and glycerol phenylbutyrate, J. Pediatr 162 (6) (2013) 1228–1234. 23324524 10.1016/j.jpeds.2012.11.084 PMC4017326

[21] Berry SA, Lichter-Konecki U, Diaz GA, , Glycerol phenylbutyrate treatment in children with urea cycle disorders: pooled analysis of short and long-term ammonia control and outcomes, Mol. Genet. Metab 112 (1) (2014) 17–24. 24630270 10.1016/j.ymgme.2014.02.007 PMC4382922

[22] Diaz GA, Schulze A, Longo N, , Long-term safety and efficacy of glycerol phenylbutyrate for the management of urea cycle disorder patients, Mol. Genet. Metab 127 (4) (2019) 336–345. 31326288 10.1016/j.ymgme.2019.07.004

[23] Kent JD, Holt RJ, Hyperammonemic crises in patients with urea cycle disorders on chronic nitrogen scavenger therapy with either sodium phenylbutyrate or glycerol phenylbutyrate, Neuropsychiatry (London) 7 (2) (2017) 131–136.

[24] McGuire BM, Zupanets IA, Lowe ME, , Pharmacology and safety of glycerol phenylbutyrate in healthy adults and adults with cirrhosis, Hepatology (Baltimore, Md). 51 (6) (2010) 2077–2085. 10.1002/hep.23589 PMC3733097 20512995

[25] Ravicti [package insert]., Horizon Therapeutics, Inc., Deerfield, IL, 11 2019.

[26] Mokhtarani M, Diaz GA, Rhead W, , Elevated phenylacetic acid levels do not correlate with adverse events in patients with urea cycle disorders or hepatic encephalopathy and can be predicted based on the plasma PAA to PAGN ratio, Mol. Genet. Metab 110 (4) (2013) 446–453. 24144944 10.1016/j.ymgme.2013.09.017 PMC4108288

[27] Thibault A, Cooper MR, Figg WD, , A phase I and pharmacokinetic study of intravenous phenylacetate in patients with cancer, Cancer Res. 54 (7) (1994) 1690–1694. 8137283

[28] Batshaw ML, Tuchman M, Summar M, Seminara J, A longitudinal study of urea cycle disorders, Mol. Genet. Metab 113 (1–2) (2014) 127–130. 25135652 10.1016/j.ymgme.2014.08.001 PMC4178008

[29] Shchelochkov OA, Dickinson K, Scharschmidt BF, Lee B, Marino M, Le Mons C, Barriers to drug adherence in the treatment of urea cycle disorders: assessment of patient, caregiver and provider perspectives, Mol. Genet. Metab. Rep 8 (2016) 43–47. 27493880 10.1016/j.ymgmr.2016.07.003 PMC4963256

[30] Longo N, Holt RJ, Glycerol phenylbutyrate for the maintenance treatment of patients with deficiencies in enzymes of the urea cycle, Exp. Opin. Orphan Drugs 5 (12) (2017) 999–1010.

[31] Waisbren SE, Stefanatos AK, Kok TMY, Ozturk-Hismi B, Neuropsychological attributes of urea cycle disorders: a systematic review of the literature, J. Inherit. Metab. Dis 42 (6) (2019) 1176–1191. 31268178 10.1002/jimd.12146 PMC7250134

[32] Soria LR, Ah Mew N, Brunetti-Pierri N, Progress and challenges in development of new therapies for urea cycle disorders, Hum. Mol. Genet 28 (R1) (2019) R42–R48. 31227828 10.1093/hmg/ddz140

[33] Yu L, Rayhill SC, Hsu EK, Landis CS, Liver transplantation for urea cycle disorders: analysis of the united network for organ sharing database, Transplant. Proc 47 (8) (2015) 2413–2418. 26518943 10.1016/j.transproceed.2015.09.020

