# Supplementary material for: Glycerol phenylbutyrate efficacy and safety from an open label study in pediatric patients under 2 months of age with urea cycle disorders
Source: Mol Genet Metab. Author manuscript; Available in PMC 2021 Dec 9. (PMC8655853; doi:10.1016/j.ymgme.2020.12.002)
Supplement: Suppl Materials [file NIHMS1759702-supplement-Suppl_Materials.docx]

**SUPPLEMENT**

**Supplementary Table 1: Schedule of assessments**

| **Time** | **Transition** | | | | | | **Safety Extension** | |  |
| --- | --- | --- | --- | --- | --- | --- | --- | --- | --- |
| **Time** | **Screen-ing ^1^** | **Day 1^1^** | **Day 2^2^** | **Day 3^3^** | **Day 4^4^** | **Day 7** | **Month 1-6 (monthly visits)** | **Months 9-24 ^5^ (Quarterly visits)** | |
| **Window (+/- days)** |  |  |  |  |  | 4 | 7 | 14 | |
| **Informed Consent** | X |  |  |  |  |  |  |  | |
| **Inclusion/Exclusion** | X | X |  |  |  |  |  |  | |
| **Physical Exam** | X | X |  |  |  |  |  |  | |
| **Medical History^6^** | X |  |  |  |  |  |  |  | |
| **UCD History^6^** | X |  |  |  |  |  |  |  | |
| **Prior Medications** | X |  |  |  |  |  |  |  | |
| **Concomitant Medications** | X | X |  |  |  | X | X | X | |
| **Weight** | X | X |  |  |  |  | X | X | |
| **Height (Length)** | X | X |  |  |  |  | X | X | |
| **Head Circumference** | X | X |  |  |  |  | X | X | |
| **Vital Signs** | X | X |  |  |  | X | X | X | |
| **Hematology and Chemistry Tests^7^** |  | X |  |  |  |  | X**^7^** | X | |
| **PK Sampling** |  | X^8^ | X^9^ | X^10^ |  | X | X | X | |
| **Ammonia** |  | X | X | X | X | X | X | X | |
| **Urine Collection (Serial or single collection)** |  | X | X^9^ | X^10^ |  | X | X | X | |
| **Assess occurrence of hyperammonemic crisis (HAC)^11^** | **HAC assessments will be completed at each visit throughout the study** | | | | | | | | |
| **Bayley III Evaluation^12^** |  |  |  |  |  |  | X | X | |
| **Dietary Assessment** | X | X |  |  |  | X | X | X | |
| **Amino acid^7^** |  | X |  |  |  | X | X^7^ | X | |
| **Genetic Testing for UCD confirmation^13^** |  |  |  |  |  |  | X^13^ |  | |
| **GPB test^14^** |  |  |  |  |  |  | X^14^ |  | |
| **Hedonic Scale^15^** |  |  |  |  |  |  | X^15^ |  | |
| **Assess need for GPB dose adjustment** |  |  |  |  |  |  | X | X | |
| **Dispense/collect GPB** |  | Start on Day 1 |  |  |  | Start Safety Extension on Day 8 | X | X | |

^1^ Screening and Day 1 procedures can be combined and need not be repeated if done within 30 days (10 days for subjects less than 2 months of age) during which the subject has been stable and controlled.

^2^ Stable subjects aged 2 months and older when they enter the study were eligible for discharge on Day 2 following 24 hrs of ammonia monitoring after complete transition to GPB. All stable subjects less than 2 months of age were eligible for discharge on Day 4, following 72 hours of ammonia monitoring after complete transition to GPB.

^3^ Subjects 2 months or older who were initially transitioned to GPB following a crisis were eligible for discharge on Day 3, following 24 hours of ammonia monitoring after complete transition to GPB.

^4^ Subjects less than 2 months of age were eligible for discharge on Day 4 following 72 hours of ammonia monitoring (including 48 hours after the first full dose) after complete transition to GPB. At least 48 hours monitoring after the first full dose of RAVICTI must have occurred in an inpatient setting. If subject was discharged at the physician’s discretion after 48 hours but prior to 72 hours of monitoring, the subject returned at approximately the 60- and 72-hour mark for evaluation and plasma ammonia measurement.

^5^ Subjects who completed a minimum of 6 months of follow-up subsequently underwent quarterly visits until 1) they reached the age of 2 years or 2) they had received GPB in the study for at least 6 months and were eligible to receive commercial GPB. All subjects under 2 months of age at enrollment received GPB until they reached the age of 2 years.

^6^ Data obtained as Medical and UCD history were based on retrospective review of medical records, hospital admission and caregiver interview. Any and all available data were recorded on the appropriate CRFs and marked NA if not available. All available ammonia, HAC, medication used for UCD, the use of G and NG tubes, methods of UCD diagnosis, family history of UCD, and other significant information were captured on the appropriate CRFs.

^7^At the Month 1 visit only, blood sample for safety labs (hematology and chemistry) and amino acid was not be collected.

^8^ Only applied to newly diagnosed subjects not presenting with hyperammonemic crisis or stable subjects currently receiving sodium phenylbutyrate and/or sodium benzoate.

^9^ Only applied to subjects presenting with hyperammonemic crisis.

^10^ Only applied to subjects less than 2 months of age presenting with hyperammonemic crisis.

^11^ If subject was experiencing a HAC or serious neurological adverse events (e.g. somnolence, lightheadedness, headache, confusion) a single PK sample was taken to check for plasma PAA levels and PAA to PAGN ratio. Any HACs during the study were recorded on the corresponding CRF.

^12^ The time points for the Bayley III evaluation were determined by subject’s age at time of enrollment:

^13^ Genetic testing for UCD diagnosis confirmation was performed within 60 days of baseline if not previously available.

^14^At the Month 3 visit, the blood sample was collected for analysis of intact RAVICTI (GPB) at selected sites. The GPB PK sample was collected 1 hour after the 2nd dose of the day during the Month 3 visit.

^15^At the Month 1 visit, palatability was assessed using Hedonic Scale.

**Supplementary Table 2: Patient demographics at baseline**

|  |  | Age <1 month (N=10) | Age 1 month to <2 months (N=6) | Total, Age 0 to <2 months (N=16) |
| --- | --- | --- | --- | --- |
| Age (months) | Mean (SD) | 0.33 (0.20) | 1.66 (0.20) | 0.83 (0.70) |
|  | Median (range) | 0.30 (0.1 – 0.7) | 1.69 (1.3 – 2.0) | 0.48 (0.1 – 2.0) |
| Race | Asian | 1 (10%) | 1 (16.7%) | 2 (12.5%) |
|  | Black or African-American | 1 (10%) | 0 | 1 (6.3%) |
|  | White | 8 (80%) | 4 (66.7%) | 12 (75.0%) |
|  | Other | 0 | 1 (16.7%) | 1 (6.3%) |
| Average Height (cm) | Mean (SD) | 51.05 (4.00) | 55.77 (5.54) | 52.82 (5.02) |
|  | Median (range) | 50.25 (45.0, 58.0) | 53.15 (51.9, 66.0) | 52.00 (45.0, 66.0) |
| Average Weight (kg) | Mean (SD) | 3.54 (0.42) | 4.54 (0.62) | 3.92 (0.70) |
|  | Median (range) | 3.58 (2.9, 4.0) | 4.50 (3.8, 5.5) | 3.87 (2.9, 5.5) |
| BMI (kg/m^2^) | Mean (SD) | 13.67 (1.69) | 14.65 (1.25) | 14.04 (1.57) |
|  | Median (range) | 14.13 (10.2, 15.8) | 14.65 (12.6, 16.2) | 14.38 (10.2, 16.2) |
| Average head circumference (cm) | Mean (SD) | 35.01 (1.18) | 37.64 (1.71) | 36.00 (1.88) |
|  | Median (range) | 35.13 (33.0, 36.5) | 37.38 (35.9, 40.8) | 35.93 (33.0, 40.8) |
| BSA (m^2^) | Mean (SD) | 0.22 (0.02) | 0.26 (0.03) | 0.24 (0.03) |
|  | Median (range) | 0.22 (0.2, 0.3) | 0.26 (0.2, 0.3) | 0.24 (0.2, 0.3) |

**Supplementary Table 3: Glycerol phenylbutyrate (GPB) Exposure during transition**

|  | Age 0 to <1 month (N=10) | | Age 1 month to <2 months (N=6) | | Age 0 to <2 months (N=16) | |
| --- | --- | --- | --- | --- | --- | --- |
|  | **Mean** | **Median (Range)** | **Mean** | **Median (Range)** | **Mean** | **Median (Range)** |
| Daily dosage, mL/day | 1.88 | 1.80 (1.3 – 2.4) | 2.20 | 2.05 (1.0 – 3.9) | 2.00 | 1.90 (1.0 – 3.9) |
| Daily dosage, mL/m^2^/day | 8.38 | 8.36 (5.8 – 10.9) | 8.42 | 8.56 (4.0 – 15.6) | 8.39 | 8.36 (4.0 – 15.6) |
| Daily dosage, mg/kg/day | 583.57 | 589.09 (400.6 – 774.3) | 534.97 | 544.58 (247.8 – 975.0) | 565.34 | 571.91 (247.8 – 975.0) |
|  | **N** | **Percentage (%)** | **N** | **Percentage (%)** | **N** | **Percentage (%)** |
| Frequency of dosing, Day 1 | | | | | | |
| BID | 1 | 10 | 0 | 0 | 1 | 6 |
| TID | 4 | 40 | 3 | 50 | 7 | 44 |
| QID | 3 | 30 | 2 | 33 | 5 | 31 |
| Other | 1 | 10 | 0 | 0 | 1 | 6 |
| QD | 1 | 10 | 1 | 17 | 2 | 13 |

**Supplementary Table 4: Plasma Pharmacokinetics Results for the First Full Day of GPB Dosing**

| **Parameter** | **PBA (N=16)** | **PAA (N=16)** | **PAGN (N=16)** |
| --- | --- | --- | --- |
| Max plasma concentration, C_max_ (μg/ml)  Mean (SD) | 46.2 (49.8) | 115.2 (102.0) | 102.1 (48.6) |
| Avg plasma concentration, C_avg_ (μg/ml)  Mean (SD) | 23.2 (23.0) | 87.0 (100.3) | 80.0 (47.3) |
| Avg exposure AUC_0-last_ (μg*h/ml)  Mean (SD) | 374.5 (390.5) | 1321.2 (1220.5) | 1384.1 (1141.0) |

**Supplementary Table 5: Concentrations of GPB analytes** **[Mean (SD)] during the safety extension**

| **Time** | **Plasma PBA (µg/mL)** | **n** | **Plasma PAA (µg/mL)** | **n** | **Plasma PAGN (µg/mL)** | **n** | **Urinary PAGN (µg/mL)** | **n** |
| --- | --- | --- | --- | --- | --- | --- | --- | --- |
| Month 1 | 15.3 (8.1) | 12 | 73.2 (68.6) | 12 | 64.6 (30.6) | 12 | 4517 (2485) | 11 |
| Month 2 | 16.6 (19.0) | 12 | 101.7 (85.1) | 10 | 53.0 (34.4) | 13 | 4116 (3137) | 12 |
| Month 3 | 30.4 (39.6) | 11 | 146.1 (207.2) | 12 | 71.4 (55.8) | 13 | 7037 (4493) | 9 |
| Month 4 | 29.1 (40.3) | 11 | 100.7 (115.1) | 10 | 48.1 (27.0) | 12 | 2826 (1543) | 9 |
| Month 5 | 44.1 (33.5) | 11 | 87.1 (114.6) | 11 | 69.5 (55.4) | 11 | 6973 (3682) | 8 |
| Month 6 | 27.1 (29.4) | 8 | 39.3 (52.8) | 8 | 40.3 (31.4) | 9 | 5883 (3128) | 3 |
| Month 9 | 21.1 (31.6) | 8 | 90.5 (78.5) | 7 | 60.7 (38.1) | 8 | 7006 (4289) | 7 |
| Month 12 | 13.4 (13.7) | 6 | 68.5 (52.3) | 5 | 58.7 (42.7) | 6 | 5847 (2992) | 3 |
| Month 15 | 31.9 (35.4) | 3 | 33.5 (40.7) | 3 | 37.2 (14.4) | 3 | 3915 (2584) | 4 |
| Month 18 | 40.7 (29.2) | 3 | 48.4 (37.8) | 3 | 73.9 (46.0) | 3 | NA | NA |
| End of treatment | 15.6 (15.0) | 9 | 50.0 (55.3) | 9 | 44.9 (35.7) | 11 | 6939 (6581) | 8 |

**Supplementary Table 6: Summary of mean (SD) Z scores for weight, height, head circumference, BSA and BMI over 12 months**

|  | Mean (SD) Z score for weight | n | Mean (SD) Z score for height | n | Mean (SD) Z score for head circumference | n | Mean (SD) Z score for BSA | n | Mean (SD) Z score for BMI | n |
| --- | --- | --- | --- | --- | --- | --- | --- | --- | --- | --- |
| Baseline | 0.21 (0.88) | 16 | 0.53 (2.19) | 16 | 0.41 (1.10) | 16 | -0.20 (2.12) | 16 | -0.05 (1.27) | 16 |
| Month 1 | -0.47 (0.92) | 16 | -0.50 (1.19) | 16 | 0.30 (1.33) | 16 | 0.04 (1.55) | 16 | -0.27 (1.24) | 16 |
| Month 2 | -0.46 (0.89) | 15 | -0.36 (1.46) | 15 | 0.16 (0.99) | 14 | -0.05 (1.53) | 15 | -0.35 (1.18) | 15 |
| Month 3 | -0.39 (0.82) | 15 | -0.34 (1.64) | 15 | -0.03 (1.11) | 14 | 0.02 (1.50) | 15 | -0.25 (1.18) | 15 |
| Month 4 | -0.41 (1.10) | 12 | -0.43 (1.59) | 12 | -0.03 (1.10) | 12 | 0.04 (1.72) | 12 | -0.20 (1.48) | 12 |
| Month 5 | -0.37 (1.08) | 11 | -0.49 (1.40) | 11 | 0.11 (1.11) | 11 | 0.05 (1.40) | 11 | -0.11 (1.35) | 11 |
| Month 6 | -0.38 (0.88) | 10 | -0.29 (2.11) | 10 | -0.05 (1.11) | 10 | -0.03 (1.96) | 10 | -0.26 (2.02) | 10 |
| Month 9 | -0.36 (1.16) | 9 | -1.13 (1.70) | 9 | -0.80 (1.12) | 9 | 0.42 (1.71) | 9 | 0.40 (1.82) | 9 |
| Month 12 | -0.25 (1.12) | 6 | -1.26 (0.92) | 6 | -1.03 (1.09) | 6 | 0.50 (1.71) | 6 | 0.63 (1.85) | 6 |

**Supplementary Figure 1: Mechanism of action of GPB**

NH_3_ = ammonia; PAA = phenylacetate; PAGN = phenylacetylglutamine; PBA = phenylbutyrate

**Supplementary Figure 2: Disposition of all patients in the study**


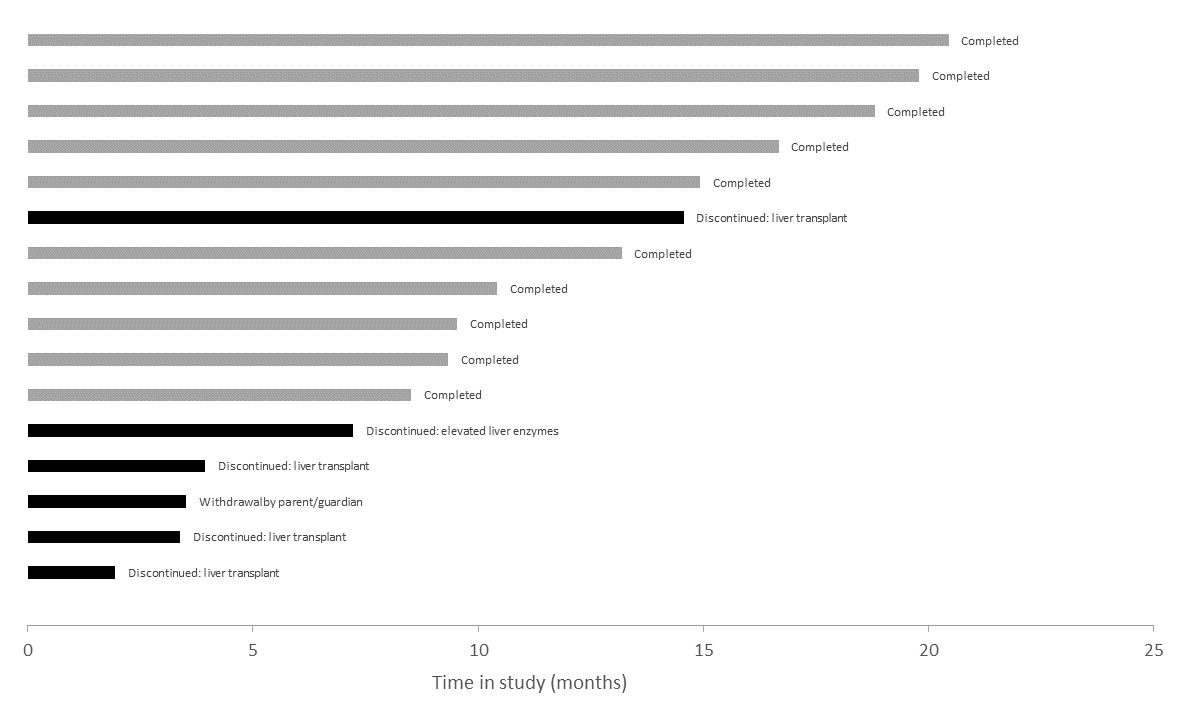


1 patient withdrew from the study prior to receiving any GPB
